# Advancing Immunization Strategies in Immunosuppressed Populations—Current Evidence, Clinical Challenges, and Future Directions

**DOI:** 10.3390/vaccines14100903

**Published:** 2026-10-15

**Authors:** Daniele Focosi, Massimo Franchini

**Affiliations:** 1 Division of Transfusion Medicine, Azienda USL Toscana Nord-Ovest, 54100 Massa, Italy; 2 Division of Hematology and Transfusion Medicine, Carlo Poma Hospital, 46100 Mantua, Italy

## Introduction

Vaccination represents one of the most effective public health interventions for preventing infectious diseases, reducing morbidity and mortality, and protecting vulnerable populations. However, individuals with impaired or dysregulated immune systems continue to represent a major challenge for contemporary vaccinology. Immunosuppressed patients—including solid organ transplant recipients, individuals receiving immunomodulatory or immunosuppressive therapies, patients with autoimmune and inflammatory diseases, and those affected by hematological or oncological disorders—often experience an increased risk of severe infections, while simultaneously demonstrating reduced or heterogeneous responses to vaccination. The balance between achieving adequate protection and ensuring safety remains a central question in clinical immunology.

The Special Issue *“Immunization of Immunosuppressed Patients”* brings together a diverse collection of original research articles and reviews addressing the complexity of vaccine responses in immunocompromised populations. Across twelve contributions [1,2,3,4,5,6,7,8,9,10,11,12], this issue highlights the evolving landscape of immunization strategies, spanning evaluation of vaccine effectiveness, characterization of humoral and cellular immune responses, optimization of vaccination schedules, assessment of specific high-risk groups, and exploration of emerging approaches to improve protection in patients with compromised immunity.

One of the major challenges addressed by several contributions is the variability of vaccine-induced immune responses among immunosuppressed individuals. Although many patients can develop measurable antibody responses following vaccination, the magnitude, quality, and durability of protection may be reduced compared with immunocompetent populations. Importantly, protection cannot be assessed solely through antibody measurements; cellular immunity, immune memory, and functional characteristics of vaccine-induced responses are increasingly recognized as essential components of effective protection. This broader immunological perspective is particularly relevant in populations where conventional serological markers may underestimate clinically meaningful protection.

Another important theme emerging from the Special Issue is the need to optimize vaccination timing and strategy in relation to immunosuppressive therapies. Many modern treatments, including targeted biological agents and cellular-depleting therapies, have transformed the management of autoimmune diseases, malignancies, and transplantation. At the same time, these therapies may substantially influence antigen presentation, B-cell and T-cell responses, and the ability to generate durable vaccine-induced immunity. Understanding how treatment schedules interact with vaccination is therefore essential for maximizing vaccine effectiveness without compromising disease control.

The contributions included in this issue also reinforce the importance of evidence-based approaches for populations that have historically been underrepresented in vaccine trials. While immunocompromised patients are among those who may benefit most from effective vaccination programs, they are frequently excluded from pivotal clinical studies. As a result, clinicians often must make decisions based on limited evidence, extrapolation from other populations, or post-marketing observational data. The studies gathered in this Special Issue contribute valuable information to address these evidence gaps and support more informed clinical decision-making.

The increasing complexity of immunosuppression in modern medicine also highlights the importance of multidisciplinary collaboration. Effective vaccination strategies require integration between vaccinologists, infectious disease specialists, immunologists, transplant physicians, rheumatologists, hematologists, oncologists, and primary care providers. Such collaboration is necessary not only to define optimal vaccine schedules but also to improve vaccine uptake, address hesitancy, and ensure equitable access to preventive interventions among medically vulnerable populations.

Together, the articles presented in this Special Issue provide a comprehensive view of the current state of immunization in immunosuppressed patients. They demonstrate significant progress while simultaneously emphasizing that important challenges remain. Improving our understanding of immune correlates of protection, refining individualized vaccination approaches, incorporating novel vaccine technologies, and expanding clinical evidence will be essential steps toward achieving more effective and durable protection for these high-risk populations.

Although the studies in this Special Issue encompass different patient groups, immune conditions, vaccine platforms, and clinical settings, several interconnected themes emerge: the need for improved characterization of vaccine-induced immunity, the importance of adapting vaccination strategies to specific immunosuppressive contexts, and the necessity of translating immunological findings into practical clinical recommendations.

A central contribution of this Special Issue is the recognition that vaccine responses in immunosuppressed patients are highly heterogeneous. Unlike healthy individuals, in whom standardized vaccination schedules generally provide predictable levels of protection, immunocompromised patients may demonstrate substantial variation in vaccine responsiveness. Several articles highlight that factors such as underlying disease, age, comorbidities, immunosuppressive treatment regimens, and the timing of vaccination relative to therapy all contribute to differences in immune outcomes. This variability reinforces the importance of individualized approaches rather than relying exclusively on uniform recommendations.

A particularly important aspect explored across the Special Issue is the evaluation of humoral immunity following vaccination. Antibody responses remain a widely used indicator of vaccine immunogenicity because they are relatively accessible and clinically interpretable. However, the contributions collected here [1,2,3,4,5,6,7,8,9,10,11,12] emphasize that antibody quantity alone does not fully capture immune protection in immunosuppressed patients. Reduced antibody titers, accelerated antibody waning, or impaired affinity maturation may occur despite the presence of clinically relevant immune responses. These observations highlight the need for complementary assessment of cellular immunity and immune memory, especially in populations where B-cell responses may be impaired.

The role of cellular immunity represents another major theme emerging from the Special Issue. T-cell-mediated responses can provide important protection against severe disease, particularly when antibody responses are incomplete or decline over time. Several studies emphasize the importance of investigating cellular immune mechanisms to better understand vaccine effectiveness in immunocompromised populations. Future clinical studies incorporating both humoral and cellular endpoints will likely provide a more comprehensive assessment of vaccine-induced protection and may help define more meaningful correlates of immunity.

Another recurring focus of the Special Issue is vaccination in patients receiving targeted immunomodulatory therapies. Advances in immune-based treatments have dramatically improved outcomes for many chronic inflammatory diseases, malignancies, and transplant-related conditions. However, these therapies can alter immune pathways essential for vaccine responsiveness. The timing of vaccination in relation to treatment initiation, treatment cycles, and immune recovery has therefore become a critical consideration. The studies presented here contribute to a growing evidence base suggesting that vaccination strategies should be integrated into broader treatment planning, ideally before profound immunosuppression occurs whenever clinically feasible.

The Special Issue also highlights the importance of studying vaccination strategies in specific clinical populations rather than considering all immunosuppressed individuals as a single group. Patients with autoimmune diseases, hematological malignancies, solid organ transplantation, and other immune disorders differ substantially in their mechanisms of immune impairment. The effectiveness and optimal timing of vaccination may therefore vary considerably between groups. By addressing distinct patient populations, the contributions in this issue help refine our understanding of disease-specific and therapy-specific factors influencing vaccine responses.

Booster vaccination and repeated antigen exposure represent another important area addressed within the broader themes of this collection. As immune responses may be reduced or less durable in immunosuppressed individuals, additional vaccine doses or modified schedules may be required to achieve adequate protection. However, the optimal approach remains an area of active investigation. Determining which patients benefit most from additional doses, when boosters should be administered, and whether immune monitoring can guide these decisions are important questions for future research.

Beyond immunogenicity, several contributions emphasize the importance of clinical effectiveness and real-world outcomes. Laboratory measures of immune response must ultimately be linked to meaningful clinical endpoints, including reduction in infection risk, and prevention of severe disease, hospitalization, and mortality. Observational studies and real-world evidence are particularly valuable in immunosuppressed populations because they capture the complexity of clinical practice and include patient groups often excluded from randomized vaccine trials.

The Special Issue also underscores the continuing need to improve vaccine implementation among immunosuppressed patients. Even when vaccines are demonstrated to be safe and beneficial, uptake may be affected by concerns regarding adverse effects, uncertainty about effectiveness, competing medical priorities, and communication challenges between patients and healthcare providers. Addressing these barriers requires not only scientific advances but also effective strategies for patient education, shared decision-making, and integration of vaccination into routine care pathways.

Collectively, the studies presented in this Special Issue demonstrate that improving vaccination outcomes in immunosuppressed populations requires a multidimensional approach. Advances in immunology, clinical research, vaccine technology, and healthcare delivery must converge to develop strategies that are both scientifically optimized and practically applicable. The evidence presented here supports a transition from generalized vaccination recommendations toward precision immunization approaches that consider individual immune status, treatment characteristics, and clinical risk.

Importantly, this body of work also highlights the progress made in recent years. The increasing availability of immune monitoring techniques, improved understanding of mechanisms underlying vaccine responses, and growing recognition of the importance of immunocompromised populations in vaccine research have substantially advanced the field. Nevertheless, significant questions remain regarding long-term protection, optimal immune correlates, vaccine platform selection, and strategies for patients with profound or persistent immune dysfunction.

The studies included in this Special Issue collectively demonstrate that vaccination of immunosuppressed patients has entered an era of increasing complexity and opportunity. While substantial progress has been achieved in understanding vaccine-induced immunity in vulnerable populations, important challenges remain. Future advances will require continued integration of clinical research, immunological investigation, and innovative approaches to vaccine development and implementation.

One of the most important priorities for future research is the identification of reliable correlates of protection in immunosuppressed populations. Although antibody responses remain valuable indicators of vaccine immunogenicity, they represent only one component of a broader immune response. Defining how antibody quantity, antibody functionality, T-cell responses, memory responses, and innate immune activation collectively contribute to protection will be essential for developing more accurate strategies to evaluate vaccine effectiveness. Such knowledge may ultimately allow clinicians to move beyond standardized vaccination schedules toward immune-guided approaches tailored to individual patients.

Another critical area of development is the optimization of vaccination strategies in relation to immunosuppressive therapies. As the therapeutic landscape continues to expand, with increasing use of targeted biological agents, small molecules, cellular therapies, and combination regimens, understanding their impact on vaccine responsiveness will become increasingly important. Future studies should focus not only on whether patients respond to vaccination but also on identifying the optimal timing, dosing, and sequence of vaccination relative to immunosuppressive treatment. Integrating vaccination planning into routine management of immune-mediated diseases and malignancies could substantially improve preventive care.

The emergence of novel vaccine platforms also offers promising opportunities for immunosuppressed populations. Advances in messenger RNA technologies, recombinant vaccine approaches, adjuvant systems, and personalized immunization strategies may provide new tools to overcome limitations associated with conventional vaccines. However, these approaches require careful evaluation in immunocompromised populations, as immunological mechanisms that determine vaccine success may differ substantially from those in healthy individuals.

Equally important is the continued expansion of clinical research participation among immunosuppressed patients. Historically, these populations have frequently been excluded from vaccine trials because of concerns regarding safety, variable immune responses, or methodological complexity. However, exclusion from research can contribute to uncertainty in clinical practice and delay evidence-based recommendations. Future vaccine studies should incorporate immunocompromised populations earlier in development programs through appropriately designed trials, long-term follow-up, and meaningful patient-centered outcomes.

Real-world evidence will continue to play a crucial role in complementing clinical trials. Large observational cohorts, immunological registries, and healthcare databases can provide valuable insights into vaccine effectiveness across diverse patient groups and treatment contexts. Combining these data sources with advanced analytical approaches, including artificial intelligence and systems immunology, may help identify predictors of vaccine response and support individualized clinical decision-making.

Beyond scientific innovation, successful immunization strategies depend on effective healthcare delivery. Ensuring that immunosuppressed patients receive timely and appropriate vaccination requires coordination among specialists, primary care providers, pharmacists, and public health systems. Education of both healthcare professionals and patients remains essential to address misconceptions, communicate realistic expectations regarding vaccine protection, and reinforce the importance of preventive care.

The COVID-19 pandemic has further emphasized the vulnerability of immunosuppressed individuals to infectious diseases while simultaneously accelerating advances in vaccinology and immune monitoring. Lessons learned from this period extend beyond SARS-CoV-2 and provide valuable frameworks for addressing other vaccine-preventable diseases in immunocompromised populations. The experience gained during the pandemic has demonstrated the importance of rapid vaccine development, adaptive vaccination strategies, and close monitoring of immune responses in high-risk groups.

In conclusion, this Special Issue provides an important contribution to the evolving field of immunization in immunosuppressed patients. The twelve articles collectively demonstrate both the progress achieved and the complexity that remains. They highlight that effective vaccination strategies must account for the diversity of immune impairments, therapeutic approaches, and patient characteristics that define modern immunosuppression.

The future of vaccination in these populations will depend on a transition toward precision vaccinology—an approach that integrates clinical characteristics, treatment history, immune profiling, and emerging technologies to deliver the right vaccine strategy to the right patient at the right time. Continued collaboration between researchers, clinicians, and patients will be essential to transform these goals into clinical practice.

We hope that this Special Issue will stimulate further investigation, encourage multidisciplinary collaboration, and contribute to improved protection against infectious diseases for individuals living with immune compromise. By advancing our understanding of vaccine responses in these vulnerable populations, the scientific community can continue to reduce preventable disease burden and improve health outcomes for patients worldwide.

## Data Availability

This manuscript generated no new dataset.
